# The miR27b-CCNG1-P53-miR-508-5p axis regulates multidrug resistance of gastric cancer

**DOI:** 10.18632/oncotarget.6374

**Published:** 2015-11-23

**Authors:** Yulong Shang, Bin Feng, Lin Zhou, Gui Ren, Zhiyong Zhang, Xing Fan, Yi Sun, Guanhong Luo, Jie Liang, Kaichun Wu, Yongzhan Nie, Daiming Fan

**Affiliations:** ^1^ State Key Laboratory of Cancer Biology & Xijing Hospital of Digestive Diseases, Xijing Hospital, Fourth Military Medical University, Xi'an 710032, China; ^2^ The 88th Hospital of PLA, Tai'an 271001, China; ^3^ Department of Plastic Surgery, Xijing Hospital, Fourth Military Medical University, Xi'an 710032, China; ^4^ Department of Ultrasound Diagnostics, Tangdu Hospital, Fourth Military Medical University, Xi'an, 710032, China

**Keywords:** gastric cancer, multidrug resistance, miR-27b, miR-508-5p, CCNG1

## Abstract

Multidrug resistance (MDR) correlates with treatment failure and poor prognosis among gastric cancer (GC) patients. In a previous study using high-throughput functional screening, we identified 11 microRNAs (miRNAs) that regulate MDR in GC and found that miR-508-5p reversed MDR by targeting ABCB1 and ZNRD1. However, the mechanism by which miR-508-5p was decreased in chemo-resistant GC cells was unclear. In this study, we found that ectopic miR-27b is sufficient to sensitize tumors to chemotherapy *in vitro* and *in vivo*. Moreover, miR-27b directly targets the 3′ untranslated regions (3′-UTRs) of CCNG1, a well-known negative regulator of P53 stability. Interestingly, miR-27b up-regulation leads to increased miR-508-5p expression, and this phenomenon is mediated by CCNG1 and P53. Further investigation indicated that miR-508-5p is directly regulated by P53. Thus, the miR-27b/CCNG1/P53/miR-508-5p axis plays important roles in GC-associated MDR. In addition, miR-27b and miR-508-5p expression was detected in GC tissues with different chemo-sensitivities, and we found that tissues in which miR-27b and miR-508-5p are up-regulated are more sensitive to chemotherapy. Together, these data suggest that the combination of miR-27b and miR-508-5p represents a potential marker of MDR. Restoring the miR-27b and miR-508-5p levels might contribute to MDR reversion in future clinical practice.

## INTRODUCTION

Cancer is the second leading cause of death worldwide, and gastric cancer (GC) remains one of the most common malignancies [1]. Chemotherapy is recommended for patients with GC who have received surgical treatment because it can prevent the recurrence or metastasis of GC. However, some patients who receive chemotherapy exhibit a poor initial response or gradually develop resistance to the chemotherapy. This phenomenon, known as multidrug resistance (MDR), might account for most cases of treatment failure among patients with GC [1–2].

For decades, researchers have explored how cancer cells acquire chemo-resistance. According to the latest studies, the expression of transporters is frequently increased in chemo-resistant cancer cells, possibly causing enhanced efflux of chemotherapeutic drugs [2–3]. Alternatively, cancer cells might antagonize the effect of drugs by altering the structure or function of drug targets. For example, the class III β-tubulin (TUBB3) isoform is insensitive to paclitaxel. GC cells overexpressing TUBB3 can survive paclitaxel exposure [4]. Reduced cell death is a hallmark of chemo-resistant cancer cells that can result from the suppression or mutation of several tumor suppressors [5–6]. The abnormal expression and function of apoptosis-associated molecules, including Bax, Bcl-2 and caspases, can promote resistance to drug-induced apoptosis [5]. Additionally, the inactivation or mutation of P53, a well-known master tumor suppressor, is associated with resistance to apoptosis [6]. Although a wide range of MDR-associated molecules has been found, the mechanisms underlying their functions and their interactions are not fully understood.

MicroRNAs (miRNAs) are a class of noncoding RNAs that can trigger either translational repression or mRNA degradation by targeting the 3′ untranslated region (3′-UTR) of specific mRNAs [7]. In a previous high-throughput functional screening study, we identified a novel subset of 11 miRNAs that was associated with MDR in GC [8]. Among these miRNAs, miR-508-5p was found to play crucial roles in regulating MDR in GC [8]. Subsequent analyses showed that each of these 11 miRNAs was associated with cellular sensitivity to at least one chemotherapeutic drug. In light of this previous research, we then examined why these 11 miRNAs, but not other miRNAs, participate in MDR in GC cells. Do these miRNAs interact or mutually regulate MDR in GC?

To examine these questions, we performed further analyses and found that miR-27b, another member of the subset of miRNAs that we newly identified, can regulate chemo-resistance and miR-508-5p expression in GC cells. Ectopic expression of miR-27b increases the chemo-sensitivity of GC cells to several chemotherapeutic drugs by inhibiting CCNG1. CCNG1 can regulate the stability of P53, a master tumor suppressor that has been shown to directly regulate miR-508-5p. We also examined the expression levels of miR-27b and miR-508-5p in GC specimens that differed in their sensitivity to chemotherapeutic drugs and found that the levels of both miR-27b and miR-508-5p were decreased in GC cases with MDR. Moreover, the miR-27b and miR-508-5p levels were evaluated in GC tissues collected from patients at different survival durations and were shown to correlate with the survival durations in patients with GC.

## RESULTS

### miR-27b regulates the chemo-sensitivity and apoptosis of GC cells

**Figure 1:**
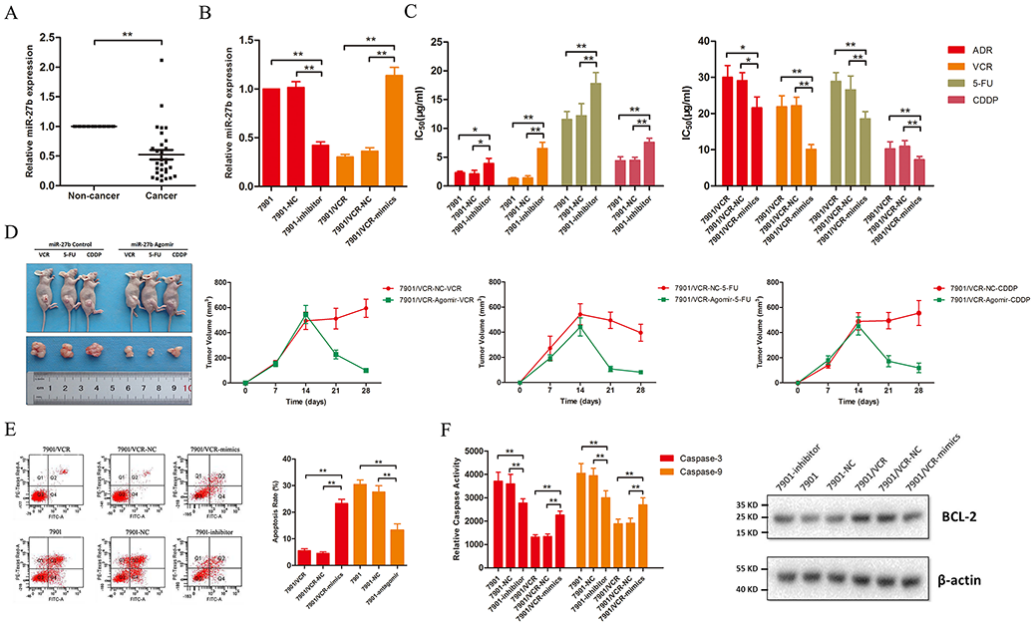
miR-27b is down-regulated in GC tissues and regulates drug sensitivity and apoptosis in GC cells **A.** The expression of miR-27b was analyzed using qRT-PCR in GC and adjacent normal tissues. **P < 0.01. **B.** Inhibitors or mimics of miR-27b were transfected into SGC7901 and SGC7901/VCR cells, respectively. **P < 0.01. **C.** Ectopic miR-27b expression sensitized SGC7901/VCR cells to several chemotherapeutic agents, whereas miR-27b knockdown increased the IC_50_ values of these drugs in SGC7901 cells. *P < 0.05, **P < 0.01. **D.** SGC7901/VCR cells were transplanted into the right flank of mice, and the mice were then treated with an agomir for miR-27b or control oligonucleotides before chemotherapy. Differences in tumor growth after chemotherapy are shown, and the tumor volumes, which were calculated as length × width^2^, were measured at the indicated time points. **P < 0.01. **E.** The apoptotic rates of cells treated with 5FU (50 μg/ml for SGC7901/VCR cells; 20 μg/ml for SGC7901 cells) for 24 h were measured via flow cytometry. **P < 0.01. **F.** The activities of caspase-3 and −9 were calculated, and Bcl-2 expression was detected by western blotting. **P < 0.01.

Previously, we identified a novel subset of 11 miRNAs that were implicated in MDR in GC. Among these candidate miRNAs, miR-27b was shown to reverse vincristine (VCR) resistance and was expressed at significantly lower levels in chemo-resistant GC cells [8]. In this study, we assessed the expression level of miR-27b in 30 paired GC and adjacent normal tissues using quantitative reverse transcriptase polymerase chain reaction (RT-PCR; qRT-PCR). As expected, miR-27b was significantly down-regulated in GC tissues (Figure 1A). To fully address the effects of miR-27b on the chemo-resistance of GC, mimics of miR-27b were transiently transfected into SGC7901/VCR cells, and an inhibitor of miR-27b was transfected into SGC7901 cells (Figure 1B). Increased IC_50_ values of the anticancer drugs adriamycin (ADR), VCR, 5-fluorouracil (5FU) and cis-platinum (CDDP) were observed after miR-27b was suppressed using inhibitors in SGC7901 cells (Figure 1C). The overexpression of miR-27b markedly sensitized cells to chemotherapy, as indicated by a decrease in the IC_50_ values of ADR, VCR, 5FU and CDDP (Figure 1C). To examine whether miR-27b regulates the drug sensitivity of GC cells *in vivo*, we transplanted SGC7901/VCR cells into nude mice. These mice were divided into 2 groups, which were treated with either agomir, which increased miR-27b expression, or control oligonucleotides. The mice that were treated with agomir exhibited markedly decreased tumor volumes after chemotherapy, indicating that drug resistance was reversed by ectopic miR-27b expression (Figure 1D).

As discussed previously, the inhibition of apoptosis is a major cause of drug resistance. Therefore, we examined whether miR-27b influences the apoptosis of GC cells. We observed that miR-27b promotes 5FU-induced apoptosis in SGC7901/VCR cells (Figure 1E). Conversely, reducing miR-27b expression decreased the apoptosis rate (Figure 1E). To further clarify how miR-27b affects apoptosis, we examined the caspase activity and Bcl-2 expression levels and found that miR-27b enhances the activity of caspase-3 and −9 and decreases the expression of Bcl-2 (Figure 1F).

### CCNG1 is a target gene of miR-27b

**Figure 2:**
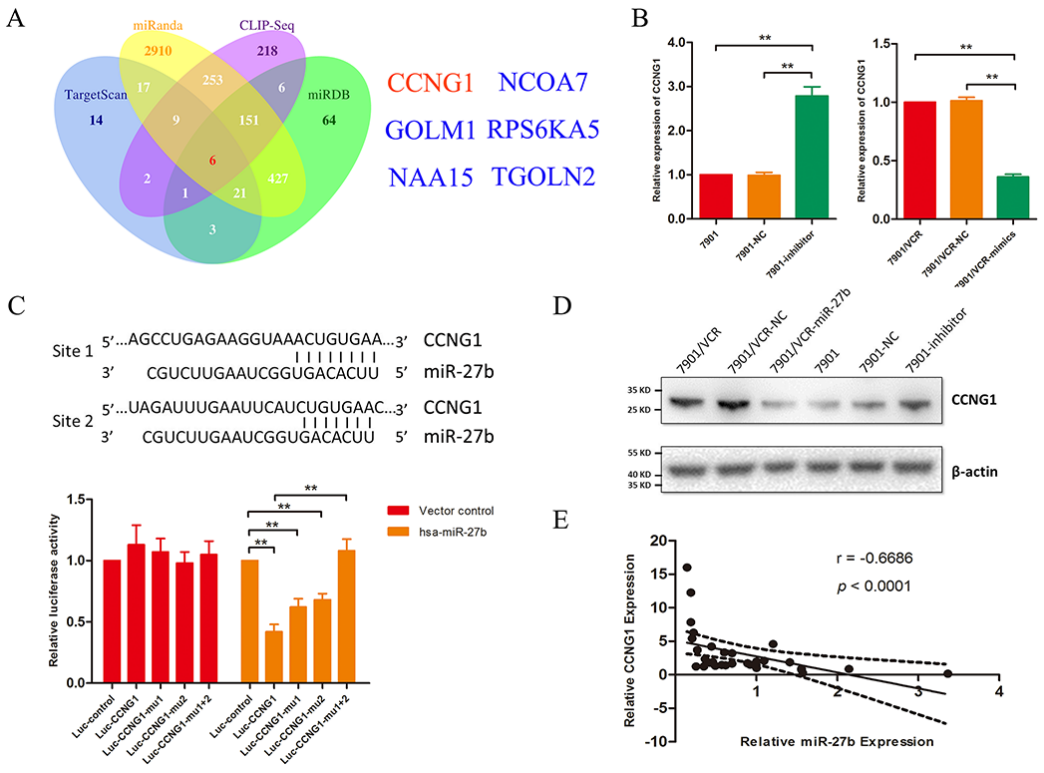
CCNG1 is a target gene of miR-27b **A.** The target genes of miR-27b were predicted using TargetScan, miRanda, miRDB and CLIP-seq. **B.** CCNG1 was detected in SGC7901/VCR cells that were treated with miR-27b mimics based on qRT-PCR. The mRNA expression of CCNG1 was analyzed following miR-27b down-regulation. **P < 0.01. **C.** The binding sites for miR-27b and CCNG1 are indicated. Luciferase assays were performed to detect the direct targeting of the 3′-UTR of CCNG1 by miR-27b. Wild-type and mutant miR-27b target sequences of CCNG1 were fused to a luciferase reporter and transfected into SGC7901/VCR cells that were stably transfected with lenti-miR-27b or its vector control. The bars represent relative luciferase activity. **P < 0.01. **D.** CCNG1 was analyzed by western blotting in GC cells in which miR-27b was transfected or inhibited. **E.** CCNG1 was examined via qRT-PCR in 30 GC tissues, and the correlation between miR-27b and CCNG1 expression was further analyzed.

Using the bioinformatics algorithms TargetScan, miRanda, miRDB and CLIP-seq, we obtained a list of candidate target genes of miR-27b. We were extremely interested in 6 genes that were identified by all 4 prediction algorithms: CCNG1, GOLM1, NAA15, NCOA7, RPS6KA5 and TGOLN2 (Figure 2A). As demonstrated above, miR-27b can regulate apoptosis. Therefore, we focused on CCNG1, which is involved in apoptosis-associated pathways, as suggested by GO analysis.

Multidrug-resistant GC cells that express miR-27b exhibit significant attenuation of CCNG1 expression at both the mRNA and protein levels (Figure 2B and 2D), whereas the inhibition of miR-27b increases CCNG1 expression (Figure 2B and 2D). Similar results were observed from tumors in mice treated with miR-27b (Supplementary Figure S1).

To assess whether CCNG1 is a direct target of miR-27b, luciferase activity assays were performed using luciferase reporters carrying the 3′-UTRs of CCNG1 (Figure 2C). In SGC7901/VCR cells that were stably transfected with the lenti-miR-27b vector or a control vector, luciferase activity was dramatically decreased. This suppression was reversed by the mutation of binding site 1 or of binding site 2 in the 3′-UTRs of CCNG1 (Figure 2C). It is well known that miRNAs can induce the degradation of target genes, and we detected a correlation between miR-27b and CCNG1 expression. As shown in Figure 2E, the expression level of miR-27b inversely correlated with the quantity of CCNG1 mRNA.

### miR-27b regulates the chemo-resistance and apoptosis of GC cells via CCNG1

**Figure 3:**
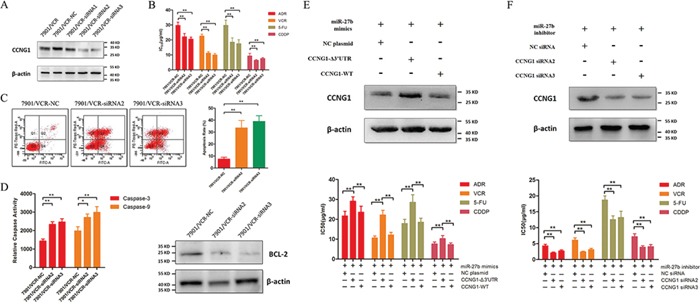
The effects of miR-27b on the chemo-sensitivity and apoptosis of GC cells are mediated by CCNG1 **A.** Western blotting of CCNG1 expression in SGC7901/VCR cells that were transfected with CCNG1-specific or negative control siRNA. **B.** IC_50_ values of ADR, VCR, 5FU and CDDP in SGC7901/VCR cells after CCNG1 knockdown. **P < 0.01. **C.** The drug-induced apoptosis of GC cells was assessed following CCNG1 knockdown. **P < 0.01. **D.** CCNG1 was silenced by siRNAs. The activities of caspase-3 and −9 and the expression of Bcl-2 were detected. *P < 0.05, **P < 0.01. **E.** SGC7901/VCR cells were co-transfected with mimics or plasmids carrying full-length CCNG1 containing or lacking its 3′-UTR. Exogenous CCNG1 antagonized the effects of miR-27b on drug resistance in SGC7901/VCR cells. **P < 0.01. **F.** SGC7901 cells were co-transfected with an inhibitor and siRNAs targeting CCNG1. CCNG1 silencing reversed the drug resistance caused by the down-regulation of miR-27b. **P < 0.01.

We examined the role of CCNG1 in drug resistance in GC. To this end, 3 pairs of siRNAs specific for CCNG1 were synthesized, and their inhibitory effects on CCNG1 were confirmed by western blotting (Figure 3A). Remarkably, silencing CCNG1 decreased the IC_50_ values of ADR, VCR, 5FU and CDDP and markedly increased the apoptosis rates when the resistant cells were treated with 5FU (Figure 3B and 3C). Knockdown of CCNG1 increased the activities of caspase-3 and −9 but suppressed Bcl-2 expression in chemo-resistant GC cells (Figure 3D).

To further confirm the role of CCNG1, we co-transfected miR-27b mimics and plasmids carrying full-length CCNG1 containing or lacking a 3′-UTR binding sites into SGC7901/VCR cells. CCNG1 lacking a 3′-UTR binding sites, but not full-length CCNG1, increased the expression of CCNG1 in cells and antagonized the effects of miR-27b (Figure 3E). Additionally, we co-transfected siRNAs targeting CCNG1 and the inhibitor of miR-27b into SGC7901 cells. We found that the effect of the miR-27b inhibitor was attenuated by the CCNG1 siRNAs (Figure 3F). These results confirmed that miR-27b regulates MDR by targeting CCNG1.

### P53 stability is promoted by miR-27b-mediated CCNG1 expression, and P53 acts as a direct modulator of miR-508-5p expression

**Figure 4:**
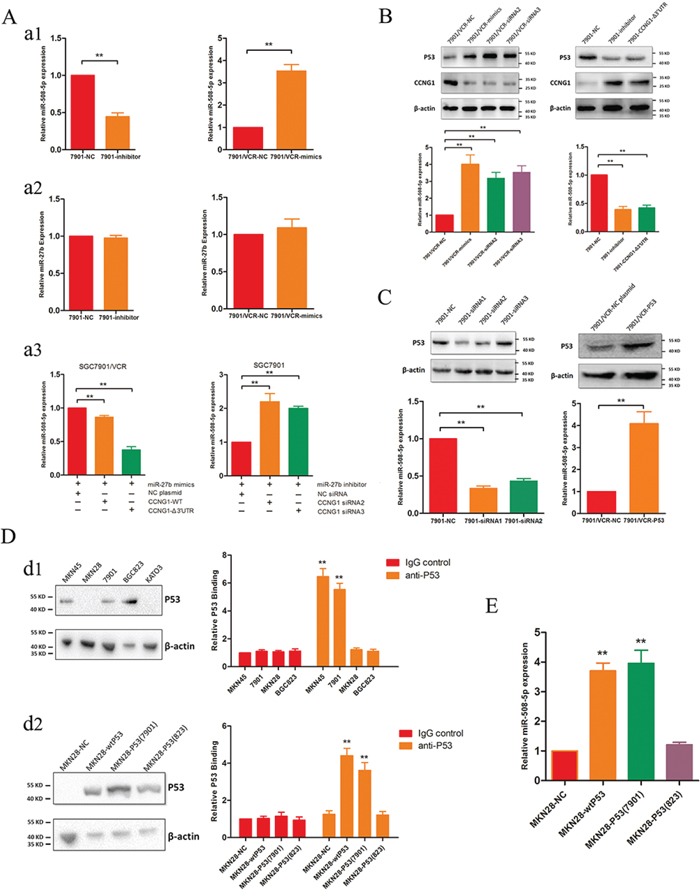
mR-27b regulates miR-508-5p via CCNG1 and P53 **A.** miR-27b regulates miR-508-5p via CCNG1. *a1*. Inhibiting miR-27b leads to decreased levels of miR-508-5p, and ectopic miR-27b expression increases the levels of miR-508-5p. **P < 0.01. *a2*. Altering miR-508-5p activity does not affect miR-27b expression. *a3*. Exogenous CCNG1 antagonizes the effects of miR-27b, and silencing CCNG1 attenuates the effects of miR-27b inhibition. **P < 0.01. **B.** CCNG1 regulates the expression levels of P53 and miR-508-5p. SGC7901/VCR cells were transfected with miR-27b or siRNAs targeting CCNG1; then, the P53 and miR-508-5p levels were detected. **P < 0.01. **C.** The expression of P53 positively correlates with that of miR-508-5p. In GC cells, P53 was modulated using siRNAs or plasmids, and miR-508-5p expression was then examined. **P < 0.01. **D.** P53 transactivates miR-508-5p in GC cells. *d1*. P53 expression was detected in several GC cell lines, and ChIP assays were subsequently performed using these cells. *d2*. Ectopic wild-type or cell line-derived P53 proteins were transfected into P53-null cells, and ChIP assays were subsequently performed. **P < 0.01. **E.** The effects of different P53 proteins on miR-508-5p expression in GC cells. **P < 0.01.

We previously found that miR-508-5p can regulate MDR in GC [8]. We were particularly interested in determining whether miR-508-5p is involved in miR-27b function. Interestingly, we found that silencing miR-27b decreases the expression of miR-508-5p in SGC7901 cells but that up-regulating miR-27b dramatically increases miR-508-5p expression (Figure 4A). Alternatively, the expression level of miR-27b remains stable regardless of the up- or down-regulation of miR-508-5p (Figure 4A). This result suggests that miR-27b might serve as an upstream modulator of miR-508-5p. To confirm whether miR-27b regulates miR-508-5p via CCNG1, we co-transfected the CCNG1-Δ3′-UTR or CCNG1-wt plasmid and a miR-27b mimic into SGC7901/VCR cells. Exogenous CCNG1 decreased the levels of miR-508-5p induced by miR-27b (Figure 4A). Moreover, RNA interference with CCNG1 siRNA ameliorated the miR-27b-induced reduction in the miR-508-5p levels (Figure 4A).

CCNG1 is reportedly a negative regulator of P53 [9]. We found that either ectopic miR-27b expression or CCNG1 knockdown increased P53 expression, whereas treatment with a miR-27b inhibitor or exogenous CCNG1 reduced P53 expression (Figure 4B). To confirm whether P53 directly regulates miR-508-5p, 3 pairs of siRNAs specific for P53 were synthesized, and siRNA1 and siRNA2 were found to effectively suppress P53 (Figure 4C). We observed that P53 siRNA transfection dramatically decreased the miR-508-5p levels but that transfection with a plasmid containing full-length P53 increased the miR-508-5p levels in SGC7901/VCR cells (Figure 4C).

To further elucidate the potential effects of P53 on miR-508-5p, we examined the expression levels of P53 in several GC cell lines. As shown in Figure 4D, abundant P53 expression was detected in MKN45, SGC7901 and BGC823 cells, but no P53 expression was observed in MKN28 or KATO3 cells. Next, we performed chromatin immunoprecipitation (ChIP) assays using MKN45, SGC7901, BGC823 and MKN28 cells. Interestingly, P53 directly bound to the promoter region of miR-508-5p in MKN45 and SGC7901 cells but not in BGC823 or MKN28 cells (Figure 4D). We constructed a wild-type P53 plasmid and directly cloned P53 from SGC7901 and BGC823 cells. These different forms of P53 were separately transfected into MKN28 cells, which lacked P53 expression, as described above. All P53 isoforms were detected in MKN28 cells after transfection (Figure 4D), and subsequent ChIP assays showed that both wild-type P53 and P53 cloned from SGC7901 cells transactivated miR-508-5p, leading to increased miR-508-5p (Figure 4D and 4E). These results are consistent with those found for endogenous P53 proteins.

Actually, mutations of TP53 occur frequently in cancer and we assumed that the sequences of TP53 might be associated with its transcriptional activities. We then performed DNA sequencing and found out the TP53 mutations in these cell lines. SGC7901 and SGC7901/VCR cells harbored the same mutations, including 140C > G in exon2, 119C > G in exon3 and 59G > A in exon4. For BGC823 cell line, it contained the same mutations as SGC7901 and SGC7901/VCR cells, and what made it different is that BGC823 cells also harbor 1 more mutation: 68C > T in exon2. Combined with the binding activities of P53 to miR-508-5p promoter from different cells, we speculated that the accumulation of more mutations might influence the core conformations that function in its binding activities, which prevented P53 from binding to the promoter of miR-508-5p.

### miR-27b and miR-508-5p are associated with chemo-sensitivity and overall survival duration among patients with GC

**Figure 5:**
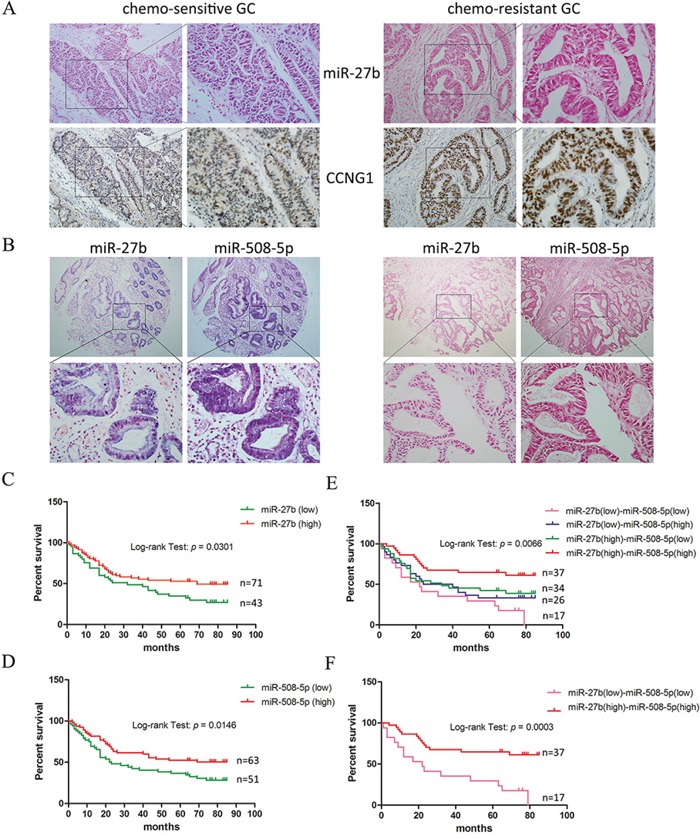
miR-27b and miR-508-5p are associated with chemo-sensitivity and the overall survival period among patients with GC **A.** The miR-27b and CCNG1 levels were examined by performing *in-situ* hybridization and immunohistochemistry on GC tissues exhibiting different sensitivities to chemotherapy. Representative samples showing an inverse correlation between miR-27b and CCNG1 expression are presented. Left panel: 200 × ; right panel: 400 ×. **B.** The miR-27b and miR-508-5p levels were detected in tissue microarrays representing 114 GC cases. Representative samples showing a positive correlation between miR-27b and miR-508-5p expression are presented. Upper panel: 40 × ; lower panel: 400 ×. **C-D.** The association between miR-27b/508-5p expression and the overall survival period among patients with GC was analyzed. **E-F.** The correlation between combined miR-27b and miR-508-5p expression and the overall survival period among patients with GC was analyzed.

To examine whether miR-27b and miR-508-5p can serve as markers of drug resistance, we analyzed the expression levels of miR-27b and miR-508-5p in GC cases displaying different chemo-sensitivities. We first collected GC tissues from 47 patients who received neoadjuvant chemotherapy before surgical treatment in our hospital between 2010 and 2012. The response to chemotherapy was evaluated according to the mRECIST criteria, and patients who were evaluated as “CR” or “PR” were regarded as sensitive to chemotherapy. Based on *in-situ* hybridization and immunohistochemical staining analyses, we found that miR-27b expression inversely correlated with CCNG1 expression in GC tissues (Figure 5A, Table 1). In addition, the levels of both miR-27b and miR-508-5p correlated with the drug responses of the GC cases. GC tissues that contained higher levels of miR-27b and miR-508-5p tended to be more sensitive to chemotherapy (Table 2). The data analysis also showed that the miR-27b and miR-508-5p expression levels were positively correlated (Table 3).

**Table 1:** The inverse correlation between miR-27b and CCNG1

	CCNG1					Spearman Test	
miR-27b	−	+	++	+++	*n*	*p*	*r*
−	1	2	3	7	47	< 0.01	−0.505
+	2	2	2	4	—	—	—
++	4	6	2	2	—	—	—
+++	5	3	2	0	—	—	—

**Table 2:** The association of miR-27b and miR-508-5p with the clinicopathologic features in GC tissues

		miR-27b					miR-508-5p				
Variables	N	−	+	++	+++	*P*	−	+	++	+++	*P*
*Age*						0.44					0.36
⊠ 60	15	2	4	6	3		5	2	3	5	
< 60	32	11	6	8	7		9	11	7	5	
*Gender*						0.83					0.83
Male	37	10	7	12	8		12	10	7	8	
Female	10	3	3	2	2		2	3	3	2	
*Differentiation*						0.71					0.60
Well - Moderate	19	6	4	4	5		7	4	5	3	
Poor	28	7	6	10	5		7	9	5	7	
*T*						0.12					0.28
T_1_–T_2_	10	5	3	2	0		4	4	2	0	
T_3_–T_4_	37	8	7	12	10		10	9	8	10	
*N*						0.28					0.60
N_0_–N_1_	28	7	4	11	6		7	9	7	5	
N_2_–N_3_	19	6	6	3	4		7	4	3	5	
*Metastasis*						0.27					0.87
M_1_	33	8	8	8	9		9	9	7	8	
M_0_	14	5	2	6	1		5	4	3	2	
*AJCC Staging*						0.68					0.68
I-II	22	7	5	7	3		7	7	5	3	
III-IV	25	6	5	7	7		7	6	5	7	
*Sensitivity*						***0.02***					***0.04***
Sensitive	13	1	1	5	6		1	3	3	6	
Resistant	34	12	9	9	4		13	10	7	4	

**Table 3:** The correlation between miR-27b and miR-508-5p

	miR-508-5p					Spearman Test	
miR-27b	−	+	++	+++	*n*	*p*	*r*
−	5	7	1	0	47	< 0.01	0.544
+	6	1	2	1	—	—	—
++	3	4	2	5	—	—	—
+++	0	1	5	4	—	—	—

Via *in-situ* hybridization assays, we detected the expression of both miR-27b and miR-508-5p in tissue microarrays representing 114 GC patients whose survival durations had been recorded. The GC cases were divided into sub-groups according to the intensities of miR-27b and miR-508-5p expression (Figure 5B, Supplementary Table S1), and their association with the overall survival duration was analyzed using Kaplan-Meier curves. Patients who expressed either miR-27b or miR-508-5p at high levels were more likely to survive than those who expressed lower miR-27b or miR-508-5p levels (Figure 5C and 5D). We then analyzed miR-27b and miR-508-5p together in our analyses and found that patients displaying different miR-27b and miR-508-p expression levels exhibited distinct survival characteristics. The overall survival duration of the GC patients simultaneously displaying elevated miR-27b and miR-508-5p levels was significantly longer than that of the remaining GC patients (Figure 5E and 5F). Taken together, these data suggest that the combination of miR-27b and miR-508-5p represents a potential marker of the chemotherapy response and the survival duration of patients with GC.

## DISCUSSION

Based on high-throughput profiling analysis, several subsets of miRNAs were suggested to be associated with drug-resistant GC [10–13], and several miRNAs were found to regulate the drug resistance of GC [10–13]. However, little research has explored whether the miRNAs that were identified by these screens are mutually associated. In a previous study, we found that the overexpression of a novel subset of 11 miRNAs, including miR-27b, reversed the MDR of GC [8]. In the present study, miR-27b was shown to participate in sensitizing GC cells to several chemotherapeutic agents. We found that the effect of miR-27b is similar to that of miR-508-5p, a miRNA that exhibits a great capacity to reverse MDR, as reported in our previous study [8]. Thus, we explored the potential association between miR-27b and miR-508-5p. Interestingly, we found that ectopic miR-27b expression leads to increased miR-508-5p expression, whereas miR-27b knockdown subsequently decreases miR-508-5p expression. These data suggest that miR-508-5p might partially contribute to the effects of miR-27b on MDR in GC.

CCNG1 is a direct target gene of miR-27b, and a loss of function study showed that the pro-apoptotic effect of miR-27b is mediated by CCNG1. CCNG1 has been identified as a homolog of c-Src kinase and has been characterized as a direct transcriptional target of P53 [9]. CCNG1 was reported to function as a suppressor of tumor proliferation in breast and lung cancer [14–15]. Several studies showed that CCNG1 plays important roles in liver cancer. CCNG1 expands liver tumor-initiating cells by inducing Sox2 via Akt/mTOR signaling, and CCNG1 was identified as a classical target of miR-122, a liver-specific miRNA [16–18]. miR-122 was found to sensitize hepatocellular carcinoma (HCC) cells to ADR and VCR by inducing cell cycle arrest, and this effect was associated with CCNG1 [17]. Further investigation showed that the miR-122/CCNG1 interaction influences p53 protein stability and transcriptional activity and reduces the invasiveness of HCC-derived cell lines [18]. Interestingly, several studies have shown that CCNG1 regulates the stability and activity of P53. For example, CCNG-deficient cells exhibit lower dephosphorylated Mdm2 levels and higher P53 protein levels than CCNG1^+/+^ cells [19–20]. Furthermore, it was observed that CCNG1-mediated P53 stabilization is dependent on Mdm2 [21]. These data strongly suggest that CCNG1 serves as a negative regulator of the P53 pathway. Indeed, we observed increased P53 expression after CCNG1 silencing, thus explaining why miR-508-5p expression increases following ectopic miR-27b expression.

In our previous study, we performed functional screening for MDR-associated miRNAs in GC. It was showed that miR-27b did not confer significant effects on cell proliferation [8]. To further confirm this, we also performed MTT assays in SGC7901 and SGC7901/VCR cells when miR-27b was down- or up-regulated (Supplementary Figure S2). The results showed that there was no significant effect of miR-27b on cell proliferation in GC. Similarly, the role of CCNG1 in cell proliferation was also studied and we found that knockdown of CCNG1 in SGC7901/VCR cells slightly suppressed cell proliferation (Supplementary Figure S2). One possible explanation to the inconsistent effects of miR-27b and CCNG1 on cell proliferation is that CCNG1 was not the only target gene for miR-27b. Therefore, the effects of CCNG1 might be attenuated by other genes targeted by miR-27b.

It has been widely suggested that P53, a master tumor suppressor protein, transactivates miRNAs but is regulated by other miRNAs [22]. In drug-resistant GC cells, P53 is suppressed by increased CCNG1 levels resulting from the down-regulation of miR-27b, and loss of P53 directly suppresses miR-508-5p. In addition, either increasing or decreasing P53 expression directly influences the expression of miR-508-5p. However, this transactivation apparently differs among P53 proteins from different cell lines. We demonstrated that endogenous or exogenous wild-type P53 regulates miR-508-5p. However, the P53 proteins from SGC7901 and BGC823 cells exhibit distinct binding activities to the promoter region of miR-508-5p. P53 from SGC7901 cells maintained the transactivation of miR-508-5p, whereas the P53 protein from BGC823 cells did not regulate miR-508-5p. Recent studies indicated that mutations were frequently observed in different GC cells and tissues [23–24]. It has been reported that mutations of P53 can suppress the function of wild-type P53 and even reverse its tumor-suppressive property [25]. The exact reasons why P53 proteins containing different mutations differentially regulate miR-508-5p remain unclear. We speculate that these different mutations might cause distinct changes to the functional domains of P53; however, further studies are needed to clarify these issues.

It has been suggested that miR-27b is associated with cell proliferation and invasion and with angiogenesis in several types of cancer; however, its function in regulating MDR remains ambiguous [26–28]. Indeed, miR-27b is reportedly up-regulated in drug-resistant sub-clones of hepatocellular carcinoma (HCC) cells, although it was not determined whether these changes in miR-27b expression directly affected the chemo-sensitivity of HCC cells [29]. However, in a recent study, miR-27b was found to be down-regulated in HCC and to affect the sensitivities of HCC cells to several therapeutic agents via p53 activation and CYP1B1 suppression [30]. The observations in this study are similar to those found in GC, suggesting that the antagonizing effect of miR-27b on MDR in cancer might depend on the P53 status. Patients carrying wild-type P53 or P53 with restricted mutations are more likely to benefit from miR-27b-targeted MDR intervention.

One of the most important aims of our study was to evaluate the potential diagnostic significance of miR-27b and miR-508-5p in GC tissues. Based on an examination of miR-27b and miR-508-5p expression in GC patients exhibiting definite drug responses, we found a direct association between miRNA expression and chemo-sensitivity. Patients with high sensitivity to chemotherapy exhibited higher expression levels of miR-27b or miR-508-5p. In addition, survival analysis of 114 patients with GC indicated that the levels of both miR-27b and miR-508-5p correlated with an extended survival duration among patients with GC. Using the combination of miR-27b and miR-508-5p might better distinguish GC patients according to their survival duration. Several miRNAs are reportedly associated with drug resistance in GC, and an attempt has been made to elucidate the miRNA signature of GC tissues following chemotherapy [10, 11, 13]. Upon the further validation of the role of these miRNAs in GC, additional study is warranted to establish a multivariate model for predicting MDR based on a panel of candidate miRNAs.

In conclusion, miR-27b is a novel miRNA that regulates MDR in GC. By targeting CCNG1, overexpressed miR-27b promotes drug-induced apoptosis and reverses GC-related MDR *in vitro* and *in vivo*. In addition, miR-27b stimulates the up-regulation of miR-508-5p by P53, and this effect is dependent on the negative regulation of P53 by CCNG1. The novel miR-27b/CCNG1/P53/miR-508-5p axis characterized here provides new insight into the mechanisms underlying drug resistance. The restoration of miR-27b and miR-508-5p expression represents a potential therapeutic strategy for the future treatment of GC patients with MDR.

## MATERIALS AND METHODS

### Cell culture and tissue collection

The human GC cell line SGC7901 and its multidrug-resistant variant SGC7901/VCR (which was established by our laboratory) were maintained in RPMI-1640 medium (Hyclone, Logan, UT, USA) that was supplemented with 10% fetal bovine serum (Gibco, Carlsbad, CA, USA), 100 U/ml penicillin sodium and 100 mg/ml streptomycin sulfate at 37°C under a humidified air atmosphere containing 5% CO_2_. Paired samples of primary GC and adjacent normal tissues were obtained from patients who had undergone surgery for GC resection at Xijing Hospital, Xi'an, China. This study was approved by the Protection of Human Subjects Committee of our hospital, and informed consent was obtained from each patient.

### RNA extraction and real-time qRT-PCR

Total RNA was extracted from cells or tissues using RNA extraction reagent (Takara Bio Inc., Otsu, Japan). To detect the expression of miR-27b, stem-loop RT-PCR was performed using an All-in-One™ miRNA qRT-PCR Detection Kit (GeneCopoeia, Rockville, MD, USA) according to the manufacturer's instructions. Primers for miR-27b and U6 were purchased from GeneCopoeia. qRT-PCR was performed to detect CCNG1 mRNA using the SYBR Green PCR Kit (Takara Bio Inc., Otsu, Japan). The mRNA level of CCNG1 was normalized to the glyceraldehyde 3-phosphate dehydrogenase (GAPDH) levels.

### Oligonucleotide construction and transfection

Mimics and inhibitors of miRNA, agomirs and negative control oligonucleotides for hsa-miR-27b were obtained from RiboBio Co., Ltd. (Guangzhou, Guangdong, China). siRNAs and plasmids for CCNG1 and TP53 were purchased from Genechem Co., Ltd. (Shanghai, China). Target cells were transfected with oligonucleotides using Lipofectamine 2000 reagent (Invitrogen) according to the manufacturer's instructions.

### *In vitro* drug sensitivity assay

A miR-27b inhibitor was transfected into SGC7901 cells, and miR-27b mimics were transfected into SGC/7901VCR cells. Drug sensitivity was assessed as described previously [8]. Briefly, 5 × 10^3^ cells were seeded in 96-well plates, and medium containing chemotherapeutic drugs was added to each well. After incubation for 48 h, a 3-(4,5-di-methyl-2-thiazolyl)-2,5-diphenyl-2H tetrazolium bromide (MTT, Sigma, St. Louis, MO, USA) assay was performed. Inhibition rates and IC_50_ values were then calculated.

### Apoptosis assay

Cell apoptosis was evaluated using an Annexin-V-FITC apoptosis detection kit (BD, Franklin Lakes, NJ, USA) as previously described [8].

### Luciferase assay

Plasmids carrying wild-type Luc-CCNG1 or mutant Luc-CCNG1-Δ3′-UTR were synthesized (GeneCopoeia, Rockville, MD, USA). The luciferase assay was performed as previously described [8].

### Western blotting

Cells or tissues were solubilized in RIPA buffer (Beyotime, China) containing protease inhibitors (Roche, Switzerland), and western blotting was performed as previously described [8]. Primary antibodies against P53 (Cell Signaling, Danvers, MA, USA), CCNG1 (Santa Cruz Biotechnology, Dallas, TX, USA) and β-actin (Sigma, St. Louis, MO, USA) were used.

### Tissue microarray, *in situ* hybridization and immunohistochemical analyses

Tissue microarray of formalin-fixed 114 GC cases (Aomei Biotechnology CoLtd, Xi'an, China) was obtained and the clinicopathologic data for each case was provided by the manufacturer. Probes (miR-27b and miR-508-5p) for *in situ* hybridization were purchased from Exiqon (miRCURY LNA detection probes, 5′-digoxigenin-labeled). *In situ* hybridization and immunohistochemical analyses were conducted as previously described [8]. All sections were examined and scored independently by two investigators in a double-blinded manner. Staining intensity was determined according to a histological scoring method. The proportion of positive cells in each specimen was quantitatively evaluated and scored as follows: 0 for staining in 0% of the cells examined, 1 for 0.01–25%, 2 for 25.01–50%, 3 for 50.01–75%, and 4 for > 75%. The staining intensity was graded as follows: 0, no signal; 1, weak; 2, moderate; and 3, strong. The histological score of the miRNAs for each section was computed using the following formula: histological score = proportion score × intensity score. A total score of 0–12 was calculated and graded as follows: negative (−, score: 0), weak (+, score: 1–4), moderate (++, score: 5–8) or strong (+++, score: 9–12). Scores of “−” and “+” were considered as low expression, whereas scores of “++” and “+++” were considered as high expression.

### *In vivo* drug sensitivity assay

Approximately 1.0 × 10^7^ SGC7901/VCR cells were subcutaneously injected into the right flank of nude mice. Mice harboring tumors were injected with the agomir or the control for miR-27b. Two weeks later, the mice were intraperitoneally injected with PBS containing VCR, 85FU8 or CDDP once weekly. The tumor volume was measured using a Vernier caliper on days 7, 14, 21, and 28 and was calculated as follows: volume = length × width^2^. The mice were humanely killed on day 28; then, the tumors were photographed. The expression levels of miR-27b and CCNG1 were analyzed using western blotting and qRT-PCR.

### ChIP

ChIP assays were performed as previously described using an anti-P53 antibody [31]. Before immunoprecipitation, 10% of chromatin was used as an input control, and a non-specific antibody (rabbit anti-IgG; BD Biosciences) served as a negative control. The precipitated DNAs were subjected to qRT-PCR in an attempt to amplify the P53-binding sites using primers that are specific for miR-508-5p.

### Caspase activity determination

The activities of caspase-3 and −9 were assessed using commercial assay kits (Beyotime Institute of Biotechnology) according to the manufacturer's instructions.

### Statistical analysis

The data are presented as the means ± the standard errors of the mean. Student's *t*-test (two-tailed) or one-way analysis of variance (ANOVA) was used to analyze the *in vitro* and *in vivo* data. A χ^2^ test was used to analyze the relationship between miR-508-5p/27b expression and clinicopathological factors. Spearman's correlation test was used to analyze the relationship between miR-508-5p and miR-27b expression. The associations between miRNAs and overall survival duration were analyzed using Kaplan-Meier curves and the log-rank test.

## SUPPLEMENTARY FIGURES AND TABLE


