# Supplementary material for: The miR27b-CCNG1-P53-miR-508-5p axis regulates multidrug resistance of gastric cancer
Source: Oncotarget. 2015 Nov 23;7(1):538–49. doi: 10.18632/oncotarget.6374 (PMC4808016; doi:10.18632/oncotarget.6374)
Supplement: Supplementary file 1 [file oncotarget-07-0538-s001.pdf]

## SUPPLEMENTARY FIGURES AND TABLE

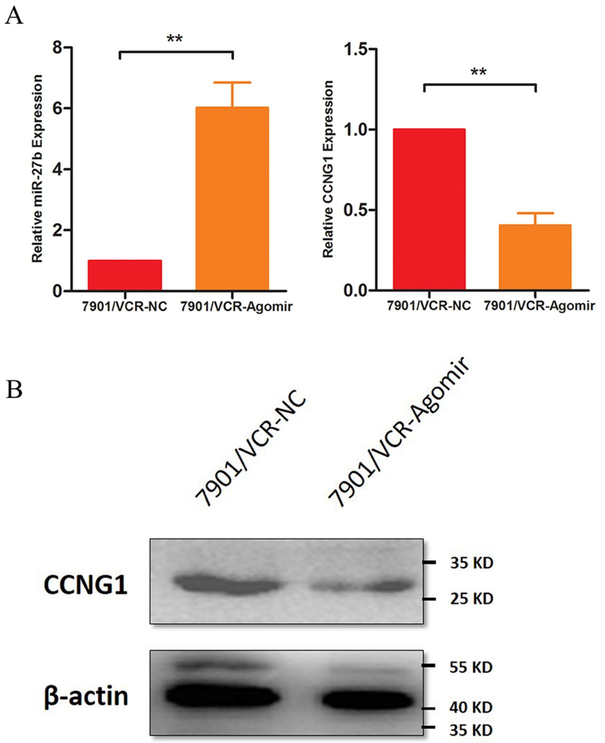


**Supplementary Figure S1: miR-27b suppresses CCNG1 *in vivo*. A.** qRT-PCR analysis of CCNG1 in tumors treated with miR-27b agomir or control. $**P < 0.01$. **B.** Western blotting analysis of CCNG1 in tumors treated with miR-27b agomir or control.

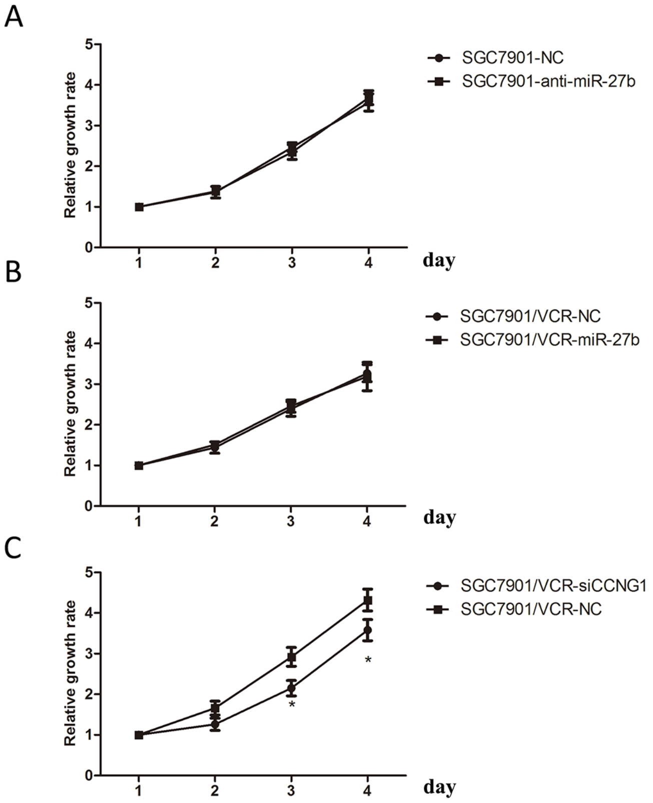


**Supplementary Figure S2: The effects of miR-27b and CCNG1 on cell proliferation. A.** Inhibitors for miR-27b was transfected into SGC7901 cells and cell proliferation was measured by MTT assays. **B.** SGC7901/VCR cells were transfected with mimics for miR-27b and MTT assays were performed to examine cell proliferation. **C.** Knockdown of CCNG1 by siRNAs in SGC7901/VCR cells suppressed cell proliferation. *$P < 0.05$.

**Supplementary Table S1: The association of miR-27b and miR-508-5p with the clinicopathologic features in GC tissues from tissue microarrays**

| Variables | N | miR-27b | | | | *P* | miR-508-5p | | | | *P* |
|---|---|---|---|---|---|---|---|---|---|---|---|
| | | – | + | ++ | +++ | | – | + | ++ | +++ | |
| *Age* | | | | | | 0.99 | | | | | 0.89 |
| ≧ 60 | 101 | 13 | 25 | 29 | 34 | | 14 | 31 | 30 | 26 | |
| < 60 | 13 | 2 | 3 | 4 | 4 | | 1 | 5 | 4 | 3 | |
| *Gender* | | | | | | 0.35 | | | | | 0.17 |
| Male | 53 | 6 | 17 | 15 | 15 | | 10 | 19 | 12 | 12 | |
| Female | 61 | 9 | 11 | 18 | 23 | | 5 | 17 | 22 | 17 | |
| *Differentiation* | | | | | | 0.61 | | | | | 0.32 |
| Well - Moderate | 40 | 3 | 10 | 13 | 14 | | 7 | 11 | 9 | 13 | |
| Poor | 74 | 12 | 18 | 20 | 24 | | 8 | 25 | 25 | 16 | |
| *T* | | | | | | 0.69 | | | | | 0.23 |
| $T_1$-$T_2$ | 15 | 1 | 5 | 5 | 4 | | 2 | 8 | 2 | 3 | |
| $T_3$-$T_4$ | 99 | 14 | 23 | 28 | 34 | | 13 | 28 | 32 | 26 | |
| *N* | | | | | | 0.11 | | | | | 0.54 |
| $N_0$-$N_1$ | 49 | 10 | 14 | 13 | 12 | | 6 | 13 | 18 | 12 | |
| $N_2$-$N_3$ | 65 | 5 | 14 | 20 | 26 | | 9 | 23 | 16 | 17 | |
| *Metastasis* | | | | | | 0.88 | | | | | 0.38 |
| $M_1$ | 10 | 1 | 3 | 2 | 4 | | 0 | 3 | 5 | 2 | |
| $M_0$ | 104 | 14 | 25 | 31 | 34 | | 15 | 33 | 29 | 27 | |
| *AJCC Staging* | | | | | | 0.23 | | | | | 0.22 |
| I-II | 48 | 9 | 11 | 16 | 12 | | 3 | 17 | 17 | 11 | |
| III-IV | 66 | 6 | 17 | 17 | 26 | | 12 | 19 | 17 | 18 | |
